# Supplementary material for: Biochemical reconstitution of branching microtubule nucleation
Source: eLife. 2020 Jan 14;9:e49797. doi: 10.7554/eLife.49797 (PMC6959992; doi:10.7554/eLife.49797)
Supplement: Transparent reporting form [file elife-49797-transrepform.docx]

***eLife’s* transparent reporting form**

We encourage authors to provide detailed information *within their submission* to facilitate the interpretation and replication of experiments. Authors can upload supporting documentation to indicate the use of appropriate reporting guidelines for health-related research (see [EQUATOR Network](http://www.equator-network.org/%20)), life science research (see the [BioSharing Information Resource](https://biosharing.org/)), or the [ARRIVE guidelines](http://www.plosbiology.org/article/info:doi/10.1371/journal.pbio.1000412) for reporting work involving animal research. Where applicable, authors should refer to any relevant reporting standards documents in this form.

If you have any questions, please consult our Journal Policies and/or contact us: [editorial@elifesciences.org](mailto:editorial@elifesciences.org).

**Sample-size estimation**

- You should state whether an appropriate sample size was computed when the study was being designed
- You should state the statistical method of sample size computation and any required assumptions
- If no explicit power analysis was used, you should describe how you decided what sample (replicate) size (number) to use

Please outline where this information can be found within the submission (e.g., sections or figure legends), or explain why this information doesn’t apply to your submission:

No statistical method was used to compute sample size when the study was designed. Sample size was determined by experimental factors. All p values and Standard Error of the Mean (SEM) values are small, which indicates that the sample size was appropriate. Experiments in Figure 1 were repeated at least three times with different Xenopus egg extracts to confirm reproducible formation of microtubule structures. Microtubule binding experiments in Figure 2 were repeated at least two times to obtain reproducible fluorescent intensity results. Branching microtubule nucleation reactions with purified augmin, TPX2 and g-TuRC in Figure 3 were repeated as stated in the figure legend to confirm reproducible results with g-TuRC purified from different preps.

**Replicates**

- You should report how often each experiment was performed
- You should include a definition of biological versus technical replication
- The data obtained should be provided and sufficient information should be provided to indicate the number of independent biological and/or technical replicates
- If you encountered any outliers, you should describe how these were handled
- Criteria for exclusion/inclusion of data should be clearly stated
- High-throughput sequence data should be uploaded before submission, with a private link for reviewers provided (these are available from both GEO and ArrayExpress)

Please outline where this information can be found within the submission (e.g., sections or figure legends), or explain why this information doesn’t apply to your submission:

Information on replicates for each experiment is included in the figure legends. No outliers were excluded.

**Statistical reporting**

- Statistical analysis methods should be described and justified
- Raw data should be presented in figures whenever informative to do so (typically when N per group is less than 10)
- For each experiment, you should identify the statistical tests used, exact values of N, definitions of center, methods of multiple test correction, and dispersion and precision measures (e.g., mean, median, SD, SEM, confidence intervals; and, for the major substantive results, a measure of effect size (e.g., Pearson's r, Cohen's d)
- Report exact p-values wherever possible alongside the summary statistics and 95% confidence intervals. These should be reported for all key questions and not only when the p-value is less than 0.05.

Please outline where this information can be found within the submission (e.g., sections or figure legends), or explain why this information doesn’t apply to your submission:

The type of test used for each dataset is included in the figure legends. Where appropriate, the legends also state that error bars are SEM. In figure 2, raw data values are plotted graphically, and n values are also explicitly stated in the figure. Exact P values are reported in the figure panels. For clarity, P < 0.00001 are reported as such.

(For large datasets, or papers with a very large number of statistical tests, you may upload a single table file with tests, Ns, etc., with reference to sections in the manuscript.)

**Group allocation**

- Indicate how samples were allocated into experimental groups (in the case of clinical studies, please specify allocation to treatment method); if randomization was used, please also state if restricted randomization was applied
- Indicate if masking was used during group allocation, data collection and/or data analysis

Please outline where this information can be found within the submission (e.g., sections or figure legends), or explain why this information doesn’t apply to your submission:

The allocation of samples into experimental groups is not relevant for this study. The experimenter was not blind to the identity of the samples during acquisition as this was not practical. However, the manual counting of microtubule branches in Figure 3E and Figure 3G, and the manual angle measurements in Figure 3F, were done blindly to prevent subconscious bias.

**Additional data files (“source data”)**

- We encourage you to upload relevant additional data files, such as numerical data that are represented as a graph in a figure, or as a summary table
- Where provided, these should be in the most useful format, and they can be uploaded as “Source data” files linked to a main figure or table
- Include model definition files including the full list of parameters used
- Include code used for data analysis (e.g., R, MatLab)
- Avoid stating that data files are “available upon request”

Please indicate the figures or tables for which source data files have been provided:

Spreadsheets with the numerical data for graphs are provided as Source data.
